# Transcriptomics reveal a unique phago-mixotrophic response to low nutrient concentrations in the prasinophyte *Pterosperma cristatum*

**DOI:** 10.1093/ismeco/ycae083

**Published:** 2024-06-14

**Authors:** Sophie Charvet, Nicholas A Bock, Eunsoo Kim, Solange Duhamel

**Affiliations:** Lamont-Doherty Earth Observatory, Columbia University, Palisades, NY 10964, United States; Division of Invertebrate Zoology, American Museum of Natural History, New York City, NY 10024, United States; Department of Biology, School of Natural and Social Sciences, Susquehanna University, Selinsgrove, PA 17870, United States; Lamont-Doherty Earth Observatory, Columbia University, Palisades, NY 10964, United States; Laboratoire d’Océanographie de Villefranche, CNRS and Sorbonne Université, 06230 Villefranche-sur-Mer, France; Division of Invertebrate Zoology, American Museum of Natural History, New York City, NY 10024, United States; Division of EcoScience, Ewha Womans University, Seoul 03760, South Korea; Lamont-Doherty Earth Observatory, Columbia University, Palisades, NY 10964, United States; Division of Invertebrate Zoology, American Museum of Natural History, New York City, NY 10024, United States; Department of Molecular and Cellular Biology, University of Arizona, Tucson, AZ 85721, United States

**Keywords:** phagotrophy, mixoplankton, bacterivory, prasinophyte, gene expression

## Abstract

Constitutive mixoplankton—plastid–bearing microbial eukaryotes capable of both phototrophy and phagotrophy—are ubiquitous in marine ecosystems and facilitate carbon transfer to higher trophic levels within aquatic food webs, which supports enhanced sinking carbon flux. However, the regulation of the relative contribution of photosynthesis and prey consumption remains poorly characterized. We investigated the transcriptional dynamics behind this phenotypic plasticity in the prasinophyte green alga *Pterosperma cristatum.* Based on what is known of other mixoplankton species that cannot grow without photosynthesis (obligate phototrophs), we hypothesized that *P. cristatum* uses phagotrophy to circumvent the restrictions imposed on photosynthesis by nutrient depletion, to obtain nutrients from ingested prey, and to maintain photosynthetic carbon fixation. We observed an increase in feeding as a response to nutrient depletion, coinciding with an upregulation of expression for genes involved in essential steps of phagocytosis including prey recognition, adhesion and engulfment, transport and maturation of food vacuoles, and digestion. Unexpectedly, genes involved in the photosynthetic electron transfer chain, pigment biosynthesis, and carbon fixation were downregulated as feeding increased, implying an abatement of photosynthesis. Contrary to our original hypothesis, our results therefore suggest that depletion of inorganic nutrients triggered an alteration of trophic behavior from photosynthesis to phagotrophy in *P. cristatum*. While this behavior distinguishes *P. cristatum* from other groups of constitutive mixoplankton, its physiological response aligns with recent discoveries from natural microbial communities. These findings indicate that mixoplankton communities in nutrient-limited oceans can regulate photosynthesis against bacterivory based on nutrient availability.

## Introduction

Mixoplankton constitute a paraphyletic assemblage of microbial eukaryotes capable of autotrophic (photosynthesis) and heterotrophic (phagotrophy, i.e. prey ingestion) nutrition [1]. With their widespread contribution to pigmented plankton communities, mixoplankton can play a significant role in marine ecosystems by fulfilling important yet underexplored ecological functions [2, 3]. For example, small-sized (2–10 μm) mixoplankton can contribute to a large fraction of bacterivory in the open ocean [4–6], implying that their absence from conceptual and biogeochemical models might lead to a mischaracterization of nutrient and carbon cycling processes [7].

Constitutive mixoplankton possess genes for both photosynthesis and phagotrophy [8]. However, while both are conserved cellular processes, the extent to which the two nutritional modes are utilized varies across different algal groups [9, 10]. Some taxa are primarily phagotrophic, using photosynthesis during periods of limited prey availability, as observed in the chrysophyte *Poterioochromonas malhamensis* [11]. In contrast, other taxa found among the prasinophytes and haptophytes are primarily photosynthetic, using phagotrophy to supplement a dietary need [12, 13]. Within this latter category, further distinctions exist between mixoplankton relying on phagotrophy for the acquisition of different resources. For instance, mixoplankton such as the haptophyte *Prymnesium parvum* [13] or the dinoflagellate *Ceratium furca* [14] obtain nutrients through phagotrophy, whereas the prasinophytes *Nephroselmis* spp. also seem to obtain vitamins from their prey [12]. While taxa such as *P. malhamensis* and *Ochromonas danica* derive carbon and/or energy from predation [11, 15], their relative *Ochromonas* sp. BG-1 can acquire both nutrients and carbon from preys [16]. In addition, some taxa, such as the dinoflagellate *Prorocentrum minimum* [17] and small marine flagellates [18], can temporarily adjust the relative contribution of each trophic mode depending on environmental conditions.

As an inducible trait, phagotrophy represents a phenotypic plasticity allowing the use of an intermittently available source of limiting elements [17] while reducing the energetic costs of maintaining two trophic modes [19]. The differential contribution of photosynthesis and phagotrophy to cellular metabolism likely depends on the availability of a growth-limiting resource, whose depletion triggers the expression of genes involved in the synthesis and activity of the cellular machinery for the alternate trophic mode. To better decipher the cellular processes involved in this shift in trophic mode, a growing number of studies have evaluated the drivers of phagocytosis in mixoplankton using comparative transcriptomics. Investigations carried out on two species of the chrysophyte genus *Ochromonas* have shown that light and prey availability have different effects on gene expression in primarily heterotrophic compared with primarily phototrophic constitutive mixoplankton [20–22]. For the primarily heterotrophic *Ochromonas* BG-1, the combined availability of light and prey led to the downregulation of photosynthesis-related genes. The same conditions stimulated upregulation of genes involved in photosynthetic processes as well as phagotrophy for the primarily phototrophic *Ochromonas* CCMP1393 [22]. These results suggest a light-dependent coupling between bacterivory and photosynthesis in primarily phototrophic mixoplankton. However, as most primarily phototrophic mixoplankton are obligate phototrophs unable to grow in the absence of light, limiting our explorations to light availability likely leads to overlooking more subtle alterations in trophic behavior that nonetheless affect carbon fixation in marine and freshwater environments.

Prasinophytes, basal members of the Chlorophyta [23], are ubiquitous in the global oceans [24–26] and some possess the capacity to ingest bacteria [12, 27–30]. In particular, members of the Pyramimonadales, such as *Cymbomonas*, *Pyramimonas*, and *Pterosperma*, were found to be bacterivorous when investigated in the laboratory [27, 28, 30] or in the field [31], which concurs with predictions from gene-based trophic models [28, 32]. Interestingly, these prasinophytes present greater bacterivory when grown under nutrient limitation [12, 28, 30, 33], a key parameter affecting the distribution of small-sized mixoplankton throughout the global ocean [34]. However, our understanding of how nutrient conditions influence the interplay between photosynthetic and phagotrophic mechanisms in bacterivorous prasinophytes remains limited. A study of *M. polaris* and *P. tychotreta* reported notable differences in their bacterivorous and transcriptional responses to nutrient depletion [33]. Still, a more recent study disputes the capacity of *M. polaris* to feed on bacteria [35]. Such discrepancies in observations within species and differences in bacterivorous activities between species underscore the necessity for further investigations into various prasinophyte lineages before we can establish a conceptual model that accurately represents all of their predatory behaviors.

To address this gap in knowledge, we compared feeding rates and gene expression in *Pterosperma cristatum* cultures exposed to different nutrient availability. *Pterosperma cristatum* NIES626 was originally collected from Seto Inland Sea, Kagawa, Japan, but this *Pterosperma* genus is globally distributed throughout the oceans [24, 36–38]. Recently identified as a mixoplankton [28], *P. cristatum* is, like most prasinophyte mixotrophs [12, 28], an obligate phototroph given its incapacity to grow under light limitation. In other obligate phototroph taxa, such as the haptophyte *P. parvum* and the dinoflagellate *Prorocentrum shikokuense,* phagotrophy provides nitrogen while cells continue photosynthetic carbon fixation for growth, as evidenced by inorganic carbon fixation measurements [13] and gene expression for this pathway [39]. Simulation models have also provided evidence that constitutive mixoplankton tend to use prey-derived resources to support photosynthetic carbon fixation instead of replacing it [40]. Hence, we hypothesized that in *P. cristatum* phagotrophy is (i) an inducible trait triggered by inorganic nutrient depletion, utilized to obtain (ii) an alternative source of nutrients to allow the cell to continue photosynthetic carbon fixation for growth. If true, we would expect that under nutrient depletion, photosynthesis-related genes would not be differentially expressed (DE) while genes related to phagotrophy would be upregulated. To test this, we examined the feeding behavior of *P. cristatum* grown in nutrient replete, nutrient reduced and nutrient depleted conditions and used comparative transcriptomics to investigate how nutrient availability affects *P. cristatum* metabolism, especially the shift between phagotrophy and phototrophy.

## Materials and methods

### Global distribution of *P. cristatum*

The distribution of *P. cristatum* was determined using MicroMap, an on-line visualization tool that uses 18S rDNA datasets to create global maps of taxon abundances [41]. The partial sequence of the 18S rDNA gene from *P. cristatum* (AB017127.3) was used as a query against the Malaspina 2010 18S-based OTUs database, with a 97% identity cutoff and an e-value threshold of 1e-10.

### Nutrient availability experiments

A uniprotistan (but xenic) culture strain of *P. cristatum* NIES626 was obtained from the Microbial Culture Collection at the National Institute for Environmental Studies (Tsukuba, Japan). The culture was maintained in f/2 medium [42] prepared with artificial seawater (made to 33 psu with Instant Ocean® Sea Salt). These pre-cultures were used to inoculate 5 replicate flasks of nutrient-replete f/2 medium and 5 replicate flasks of 10-times diluted f/2 medium (hereafter f/2 and f/20, respectively) for an initial density of 1.8 × 10^5^ cells/mL. For 18 days, the cultures were sampled every 4–5 days to determine cell abundance and feeding frequency. To evaluate the effect of nutrient availability on the metabolism of *P. cristatum*, we compared the transcriptomes of *P. cristatum* under three distinct physiological states at different nutrient availability. We identified time points where feeding frequency was increasing in the f/20 cultures compared to f/2 (Day 11) and/or was significantly different in f/20 cultures compared to the other days (Day 16) to collect RNA from all replicates of each physiological state. More detailed information on RNA sample processing can be found in Supplementary Materials.

### Bacterivory measurements

Bacterivory was evaluated by observing the ingestion of fluorescently labeled bacteria (FLB) by algal cells over 50 minutes, in 0.5 mL aliquots taken from the nutrient availability experiment growth flasks. FLB were prepared by labeling cultures of *Pelagibaca bermudensis* HTCC2601 with CellTracker Green CMFDA (Thermo Fisher Scientific, Waltham, MA), according to Bock et al. ([28]; see Supplementary materials). Supernatant from the final wash step was filtered on 0.2-μm-pore filters and saved for use as a negative control (called unfed control). To determine feeding frequencies, a Guava EasyCyte Mini Cytometer (Millipore) and its custom software guavaSoft were used to evaluate the proportion of algal cells that increased in green fluorescence following inoculation with FLB. To minimize variability in FLB encounter rates, FLB were added at a fixed proportion of 20% of the total bacterial density [43], measured by labeling a subsample with SYBR Green I (Lonza) and counting with flow cytometry. The feeding threshold was defined as the maximal green fluorescence of algal cells immediately following inoculation. The number of cells exceeding the feeding threshold was then determined at 10-minute intervals up to 50 minutes. To account for differences in the number of algal cells between replicates and treatments, the number of cells exceeding feeding thresholds was then normalized to the total number of algal cells for each time point as per_fed_. To account for any change in fluorescence due to the uptake of activated dye in the FLB matrix, unfed controls consisted in separate aliquots of f/20 cultures inoculated with a volume of FLB supernatant equal to the volume of FLB cell suspension added to f/20 cultures. Feeding frequency in these unfed controls was 0.01% min^−1^ ± 0.09. Multiple regressions comparing percentage of algal cells (per_fed_) to the time since inoculation with FLB for each treatment (per_fed_ = time ^*^ treatment) explained 97% of variability in per_fed_ (F-test; F-score = 470, df = 68, *P* < 2 × 10^−16^). More detailed information on statistics can be found in Supplementary materials.

### Dissolved inorganic nutrients

Dissolved nitrate and nitrite (NOx) and phosphate concentrations were determined by collecting 10 ml samples on Day 0 and Day 11 of experimental growth, for both f/2 and f/20 treatments, and additionally at Day 16 for the f/20 treatment. The volume was filtered through acid-washed, pre-combusted GF/F filters (MilliporeSigma) to collect the filtrate and stored at −20°C until analysis on an AutoAnalyzer AA3 HR (Software version 6.10; SEAL Analytical, Mequon, WI), as specified by the manufacturer, using multitest MT19 methods G-297-03 Rev 4 for phosphates and G-172-96 Rev 16 for nitrate.

### Transcriptome analysis

The total *P. cristatum* RNA extracts collected from three different nutrient conditions were sent to GeneWiz for the library preparations and sequencing on the Illumina HiSeq 4000 platform. The resulting reads were used to de novo assemble the algal transcriptomes (Supplementary materials Table S1) and to conduct differential gene expression analyses. For the latter, transcript counts were imported into R [44] using the tximport package [45], for subsequent differential gene expression analysis between the three experimental treatments, with DESeq2 [46] using the apeglm package for LFC shrinkage [47]. Genes were qualified as DE when the log-fold change compared to the Replete reference was >|0.1| and the adjusted *P*-value < .005. Additional details on transcriptome assembly and differential gene expression analyses are provided in Supplementary materials.

### Probing the gene-based predictive model

To identify *P. cristatum* genes that correspond to the 474 proteins identified by Burns et al. [32] as predictive of a phagocytotic capacity, a hidden Markov model search was performed as per the author’s instructions on github (burnsajohn/predictTrophicMode). The top 75% of expressed genes were then selected and annotated with the KEGG ontology, as described in Supplementary materials.

## Results

### Distribution of *P. cristatum*

*Pterosperma cristatum* was identified from surface waters sampled during the Malaspina 2010 expedition [48], which collected samples from globally distributed tropical and subtropical stations (Supplementary materials Fig. S1). 18S rDNA amplicon sequence variants related to *P. cristatum* (>97% identity) were detected in surface waters at 116 out of the 289 stations. These variants represented an average of 0.026% (ranging from 0.001 to 0.381%) of the total 18S rDNA amplicon sequences at these stations.

### Growth and feeding response to nutrient depletion

**Figure 1:**
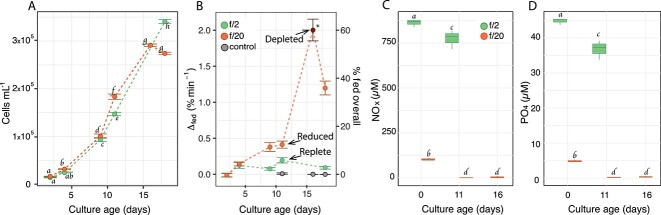
Physiological properties of *P. cristatum* cultures and nutrient concentrations. Cell abundance (*n* = 5; A) and feeding frequency (Δfed; *n* = 5; B), NOx concentrations (NOx; *n* = 3; C), and phosphate concentrations (PO4; *n* = 3; D) of *P. cristatum* in f/2 and f/20 medium over time. Error bars represent the standard deviation; italic letters indicate significant differences according to a two-way ANOVA followed by a Tuckey’s test (*P-adj* < .05); “control”, unfed control for the feeding frequency; “Replete,” nutrient replete reference; “Reduced,” nutrient reduced; “Depleted,” nutrient depleted, and arrows indicate the samples from which RNA was collected.

Cell abundances were not significantly different between treatments during the first 4 days of growth. At days 9 and 11 abundances were significantly higher in f/20 cultures than in f/2, and then at day 18, cell abundances in f/20 dropped significantly below those in f/2 (*P*-values < .05; Fig. 1A). Bacterial growth in all culture flasks (Supplementary materials Fig. S2) indicated that *P. cristatum* cells were never prey-limited. Feeding experiments showed a low baseline feeding frequency in cultures grown in f/2. The f/20 cultures tended to have higher feeding frequencies compared to the f/2 treatment, starting at day 8 (Fig. 1B). The proportion of feeding algae in the f/20 treatment was higher at days 8 and 11 (13%) compared to the f/2 replete reference (6%) and the unfed control (0%), although the feeding frequencies were not significantly different (Fig. 1B, Supplementary materials Table S2). By day 16 in f/20, however, when the proportions of feeding cells reached 60%, feeding frequencies were significantly greater than in the unfed control, and the f/2 and f/20 treatments on day 11 (Fig. 1B, Supplementary materials Table S2).Fig. Nutrients were analyzed to confirm the effects of dilutions and growth on nitrate and phosphate availability. Nitrate and phosphate concentrations at day 0 were about 10 times lower in the f/20 treatments than in the f/2 treatments (Fig. 1C, D). After 11 days of growth, nutrient concentrations remained high in the f/2 treatments while in f/20 over 90% of both nitrate and phosphate had been removed. At day 16, the cultures in f/20 treatments displayed nitrate concentrations at or below detection limits (except for one replicate with 11 μM) and phosphate concentrations were the same as on day 11. Therefore, the f/20 cultures grown beyond day 11 were considered nutrient depleted.

### *P. cristatum* transcriptome and comparative analyses

RNA was collected from each experimental replicate (*n* = 5) at day 11 from f/2 cultures (Replete reference; hereafter Replete) and f/20 cultures (nutrient Reduced condition; hereafter Reduced), and at day 16 from f/20 cultures (nutrient depleted condition; hereafter Depleted), respectively. The Replete (f/2) had relatively high nutrient concentrations and negligible feeding frequencies. The Reduced had low nutrient concentrations but growth rate was similar to the Replete, and feeding frequencies were higher. The Depleted had undetectable nutrient concentrations and high feeding frequencies, with maintained *P. cristatum* growth.

The three physiological states of *P. cristatum*, corresponding to the Replete, Reduced and Depleted nutrient conditions, provided distinct transcriptomes (Supplementary materials Fig. S3). For an assembly of 72 305 transcripts total, 52 608 (72.7%) were predicted to bear protein-coding genes (Supplementary materials Table S2), of which 10 543 were annotated with the KEGG database. 7973.8 (±574.5) were found in the Replete, 8018.2 (±586) in the Reduced and 7608.4 (±777.3) in the Depleted conditions, with a total of 5031 genes shared by all three transcriptomes. A principal component analysis of the three transcriptomes showed that most of the variability (PC1, 93%) existed between the Replete and the two nutrient-restricted conditions (Reduced and Depleted), except for one replicate of Reduced (Supplementary materials Fig. S3). This odd replicate was nonetheless retained for further analyses as its removal did not alter the results.

**Figure 2:**
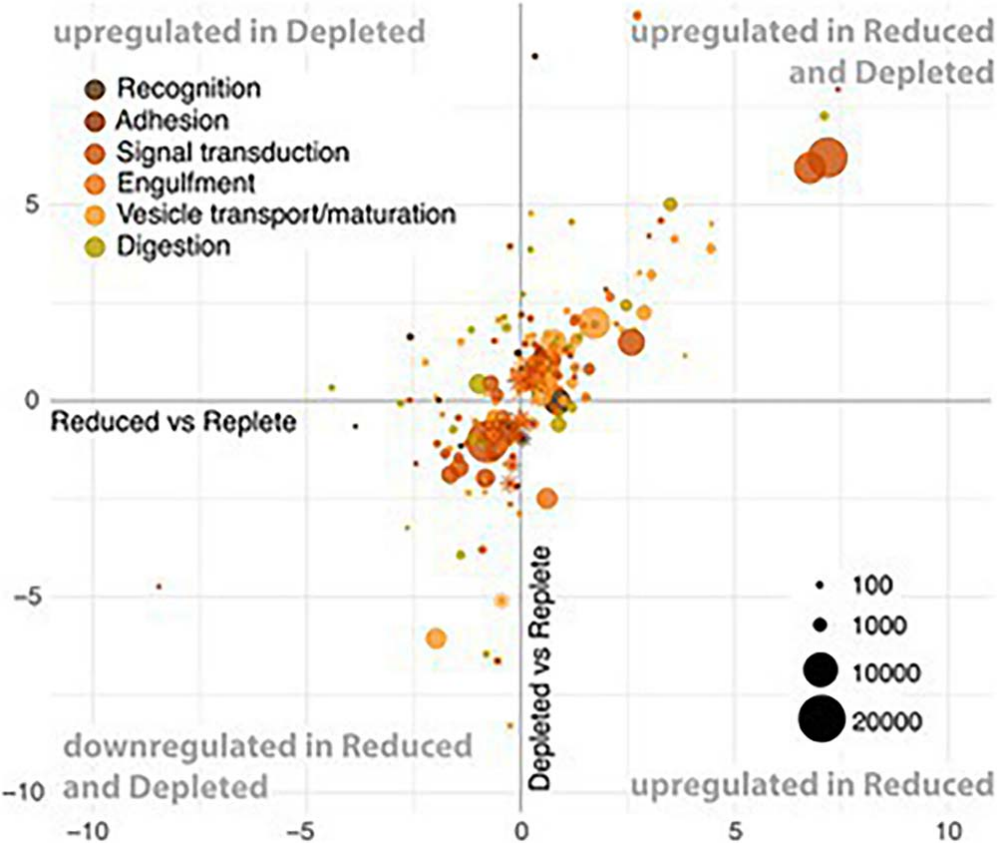
Log-fold changes plots showing DE genes involved in phagotrophy. The *x*-axis represents the log-fold change of gene expression between the nutrient reduced condition and the nutrient replete reference. The *y*-axis represents the log-fold change of gene expression between the nutrient depleted and replete reference. Genes in the lower right quadrant are upregulated in Reduced and those in the upper left quadrant are upregulated in Depleted. Genes in the upper right quadrant are upregulated in both Reduced and Depleted while those in the lower left quadrant are downregulated in both. The dot size is proportional to the average expression level of the gene. Asterisks designate genes that were significantly DE in Depleted but not Reduced.

**Figure 3:**
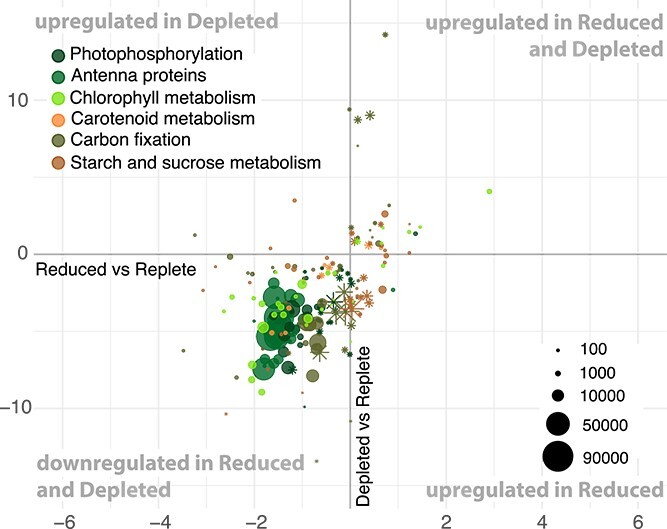
Log-fold changes plots showing DE genes involved in photosynthesis. The *x*-axis represents the log-fold change of gene expression between the nutrient reduced condition and the nutrient replete reference. The *y*-axis represents the log-fold change of gene expression between the nutrient depleted and replete reference. Relative regulation of genes per treatment is as described in Fig. 2. The dot size is proportional to the average expression level of the gene. Asterisks designate genes that were significantly DE in Depleted but not Reduced.

**Figure 4:**
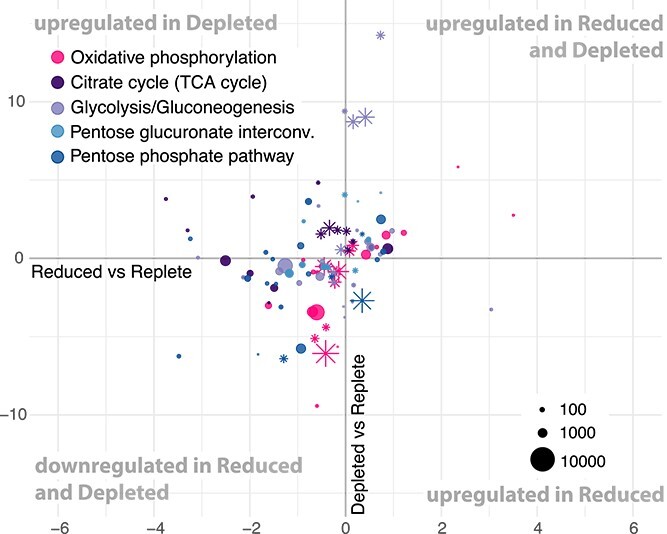
Log-fold changes plots showing DE genes involved in central carbon metabolisms. The *x*-axis represents the log-fold change of gene expression between the nutrient reduced condition and the nutrient replete reference. The *y*-axis represents the log-fold change of gene expression between the nutrient depleted and replete reference. Relative regulation of genes per treatment is as described in Fig. 2. The dot size is proportional to the average expression level of the gene. Asterisks designate genes that were significantly DE in Depleted but not Reduced.

Differential expression analysis revealed that cultures in the Depleted condition had a stronger upregulation response for a greater number of genes than the Reduced condition (Table 1). More specifically, in Reduced transcriptomes 10.3% of genes were significantly DE (5.4% upregulated and 4.9% downregulated) compared to Replete transcriptomes (Table 1, Supplementary materials Fig. S4A). In Depleted, 20.4% of genes were significantly DE compared to Replete (9.4% upregulated and 11% downregulated) (Table 1, Supplementary materials Fig. S4B). Between the Depleted and Reduced treatments, 11.1% of the genes were significantly DE (Table 1, Supplementary materials Fig. S4C). To simultaneously visualize the differential expression patterns for all three culture conditions, we plotted the log-fold change of significantly DE genes in the two nutrient-restricted conditions compared to Replete (Figs 2–4). Hereafter, any mention of DE genes pertains to comparisons to the Replete.

**Table 1:** Results of the differential expression analysis using DESeq2; DE genes in nutrient reduced (“Reduced”) and nutrient depleted (“Depleted”) sampling conditions compared to the Replete reference transcriptome; UP, upregulated proportion of DE genes; DOWN, downregulated proportion of DE genes.

	**Reduced vs Replete**			**Depleted vs Replete**			**Depleted vs Reduced**		
	Total DE	UP	DOWN	Total DE	UP	DOWN	Total DE	UP	DOWN
Absolute count	7420	3890	3530	14 697	6772	7925	7997	3458	4538
Percentage of total transcripts	10.3%	5.4%	4.9%	20.4%	9.4%	11%	11.1%	4.8%	6.3%

### Upregulation of genes involved in phagotrophy under reduced and depleted nutrient conditions (Fig. 2)

The cellular processes involved in phagocytosis of most eukaryotes correspond to (i) recognition of prey, (ii) adhesion for capture, and (iii) signal transduction, followed by (iv) engulfment, (v) transport and maturation of the phagosome, and finally (vi) digestion of its contents [49–51]. Although phagotrophy in prasinophytes might involve slight deviations from these processes [27], we looked for corresponding pathways in the KEGG ontology, including membrane receptors (ko04030, ko04054) and signal transduction (ko09132), cytoskeleton (ko04812), autophagy (ko04140, ko04138, ko04136), endocytosis (ko04144), phagosome (ko04145), lysosome (ko04142), peroxisome (ko04146) and ion transporters (ko02000, ko02010) (Table 2, Supplementary materials Table S3). Genes involved in these processes were DE in either one or both nutrient restriction treatments (Fig. 2).

**Table 2:** Overview of metabolic pathways and their representation among the DE genes in nutrient reduced (“Reduced”) and nutrient depleted (“Depleted”) sampling conditions compared to the replete reference transcriptome. UP, upregulated proportion of DE genes; DOWN, downregulated proportion of DE genes.

			**%DE in Reduced**			**%DE in Depleted**		
**Category**	**Pathway**	**Total genes**	Total DE	UP	DOWN	Total DE	UP	DOWN
Central carbon metabolism	Resp. electron transp. chain	135	8.89	4.44	4.44	19.26	13.33	5.93
	TCA cycle	66	15.15	3.03	12.12	25.76	19.70	7.58
	Glycolysis/gluconeogenesis	124	16.94	4.84	12.10	27.42	10.48	16.94
	Pentose and glucuron. interconv.	42	7.14	0.00	7.14	23.81	11.90	11.90
	Pentose phosphate pathway	69	23.19	4.35	18.84	24.64	4.35	20.29
Phagotrophy	Autophagy	67	19.40	14.93	4.48	52.24	44.78	7.46
	Lysosome	75	20.00	8.00	12.00	26.67	16.00	10.67
	Phagosome	66	7.58	4.55	3.03	21.21	9.09	12.12
	Endocytosis	83	15.66	10.84	4.82	31.33	21.69	9.64
	Cytoskeleton proteins	316	10.44	6.33	4.11	23.42	14.24	9.18
Photosynthesis	photophosphorylation	68	29.41	1.47	27.94	58.82	1.47	57.35
	Antenna	34	23.53	2.94	20.59	32.35	0.00	32.35
	Chlorophyll biosynthesis	105	19.05	4.76	14.29	30.48	5.71	24.76
	Carotenoid biosynthesis	14	35.71	7.14	28.57	50.00	7.14	42.86
	Carbon fixation	76	27.63	1.32	26.32	59.21	17.11	42.11
	Starch and sucrose metabolism	144	13.89	5.56	8.33	21.53	5.56	15.97
Other	Lipid metabolism	464	18.75	9.48	9.27	30.60	15.09	15.52
	Amino acid metabolism	744	22.45	9.54	12.90	31.72	16.26	15.46

Recognition of prey requires membrane receptors for chemosensory detection, with signal transduction to stimulate motility and ingestion [51, 52]. We identified 12 DE surface receptors in the *P. cristatum* transcriptome (Supplementary materials. Table S3), including six G-protein coupled receptors (*CASR, FOLR, GCR1, GPR3, S1PR1, ADIPOR*), of which three were upregulated in both nutrient restriction conditions (GPR3, S1PR1, ADIPOR). Adhesion molecules were also DE (Supplementary materials Table S3), with downregulation of two genes (*FAT4, LAMC1*) and the upregulation of two other genes (*ROBO1, GLG1*) in Reduced condition. In the Depleted condition, we observed the downregulation of five genes (*FAT4, LAMC1, COL1A, THBS2S, RAC1*) and the upregulation of seven (*ROBO1, GLG1, MAEA, COL4A, CNTNAP2, MEMO1, PTPRF*). Signaling pathways also responded to the lack of nutrients, with almost twice as many DE genes in Depleted than in Reduced. These pertained to the calcium, MAPK, and mTOR signaling pathways, as well as the Wnt, PI3K-Atk, FoxO, HIF-1, TGF-beta, and sphingolipid signaling pathways, showing a variety of up- and downregulated genes that intersect with other signaling pathways. Overall, we observed a tight transcriptional regulation of signal transduction by *P. cristatum* under nutrient reduction and depletion.

Engulfment was represented by twice as many upregulated genes in Depleted compared to Reduced (Supplementary materials Table S3). These were involved in food vacuole formation [53, 54], such as clathrin (*CLTC*) and ADP-ribosylation factor GTPase (*ARF1_2*). The concurrent differential expression of regulators of Arf1, namely Arf-GAP (*ACAP, ARFGAP1*) and Arf-GEF (*ARFGEF*), suggests that *P. cristatum* was regulating engulfment [55]. This was further supported by upregulation of *PIP5K*, which codes for an enzyme that produces a phosphoinositide that modulates actin polymerization [56, 57]. The transport of food vacuoles was indicated by the upregulation of Rab GTPases (*RAB11FIP3_4, RAB5C* and *RAB1A*), which are cargo markers [58], as well as microtubule assembly (*TUBA, TUBB, TTLL, TBCB*) and molecular motors belonging to the kinesin (*KIF5, KIFC1, KIFC2_*3), dynein (*DNAH, DNAAF1, DYNLL*) and myosin (*MYO1, MYO5, MYO18, MYLK, MYH1s, MYH9s, MYH6_7*) families. Maturation of food vacuoles was indicated by upregulation of CHMP genes (*CHMP1, CHMP4A_B, CHMP5*), belonging to the ESCRT-III complex [59, 60], and homologs of autophagy proteins (*ATG8, Rab1A, MON1, NAPA, VPS8* and *ZFYVE1*), likely shared with phagosome processing steps [61].

Revealing increasingly active phagocytic digestion as nutrients became more limiting, the *P. cristatum* lysosome pathway was 8% and 16% upregulated in Reduced and Depleted, respectively. Lysosomal hydrolases, for the degradation of lipids, sugars, and proteins, showed variable differential expression. A sulfatase, arylsulfatase B (*ARSB*), and two cysteine proteases, cathepsins F and X, as well as a serine/threonine-protein kinase and endoribonuclease (*ERN1*) were upregulated in both nutrient restrictions, as well as lysosomal membrane transporters (*MCOLN1, ABCA2*). Additional cathepsins (*CTSB*, *CTSD*), a peptidase (*TPP1*), and a lipidase (*ACOX1*) were also upregulated in Depleted, but other hydrolases (*CTSB, CTSC*, *LYPLA3*, *MANBA*) were downregulated. Proton transporters (*SLC36A*), metal ion transporters (*copA*), and enzymes involved in the production or transport of reactive oxygen species (*NOS2, DAO, DUOX, PEX13, SOD*) were DE under nutrient restriction, with more consistent upregulation in Depleted. These transporters were likely involved in the acidification of the food vacuole and the transport of heavy metals (Zn, Cu) and reactive oxygen species for the digestion of prey [49]. Overall, the phagotrophic processes were more profoundly impacted in Depleted, with 77% of upregulated genes being more strongly DE in Depleted than Reduced (Supplementary materials. Table S3).

To further highlight key genes in the transcriptome whose expression levels affect bacterivory, we carried out an analysis of the transcriptomes against the predictive protein set identified by Burns et al. [32]. Of the 474 phagocytic predictive protein-coding genes shared among free-living phagocytes, 67 were present in the *P. cristatum* transcriptome, and this was sufficient for phago-mixotroph predictions for all three conditions. Thirteen of these genes were annotated with the KEGG ontology, 5 of which were DE (Supplementary materials. Fig. S5). These involved a calcium channel (*CACNA1D*), a homolog of purine permease (*pbuG copA*), cytoskeleton-associated proteins and hydrolases, as well as a Bardet–Beidl Syndrome complex subunit (*BBS2*). The expression levels for these DE phagocytosis prediction genes increased in the Reduced compared to the Replete, and even more in the Depleted treatments.

### Downregulation of genes involved in photosynthetic metabolic pathways under nutrient restrictions (Fig. 3)

The cellular processes that support photosynthesis include photophosphorylation (photosynthetic electron transfer chain, PETC; ko00195), pigment biosynthesis (ko00860, ko00906) and supporting antenna proteins (ko00196), carbon fixation (ko00710) and starch production (ko00500). The genes coding for the thylakoid-bound proteins involved in the PETC were 29.4% and 58.8% DE in Reduced and Depleted respectively, and 95–97% of these genes were downregulated (Table 2, Supplementary materials Table S4). This included subunits of photosystems I (*psaD, psaF, psaH, psaL, psaO*) and II (*psbO, psbP, psbQ, psbR, psbY*), as well as the electron acceptors, plastocyanin (*petE)*, ferredoxin (*petF*), and the cytochrome b6f complex subunit Rieske Fe-S protein (*petC*). The downregulated genes for antenna proteins corresponded to subunits of light-harvesting complexes I and II (*LHCA1,2,4,5*, and *LHCB1* to *LHCB7*). In addition, the metabolism of terpenoids and porphyrins, involved in the biosynthesis of carotenoids and chlorophylls, respectively, also exhibited downregulation under both nutrient restrictions.

Genes involved in carbon fixation were 27.6% and 59.2% DE in Reduced and Depleted, respectively (Fig. 3, Table 2). Genes from the Calvin cycle were all downregulated under nutrient restrictions (*ALDO, SBPase, FBP, GAPA, glpX, GPT, PGK, rbcS, rpiA, TPI*) and two out of five genes from the C4-dicarboxylic acid cycle were downregulated (*pckA, GPT*). In addition, starch and sucrose metabolism were 13.9% and 21.5% DE in Reduced and Depleted, respectively (Supplementary materials Table S4). Four genes were downregulated (*scrK, BAM, SPP, TPS*) and three genes were upregulated (*cd, PYG, BAM*) in both nutrient restrictions. In the Depleted, an amylase (*AMY*) had increased expression while starch synthase was downregulated. These expression patterns suggest the degradation of storage sugars and the downregulation of their synthesis pathways. Overall, the photosynthetic pathways were more profoundly impacted in Depleted, with 93% of downregulated genes being more strongly DE in Depleted than in Reduced (Supplementary materials Table S4).

### Differential expression of genes involved in respiration and central carbon metabolism (Fig. 4)

To further investigate the alga’s potential metabolic shift, due to the apparent downregulation of photosynthetic carbon fixation, we further focused on the respiratory electron transport chain (RETC) and central carbon metabolism. Both pathways revealed differential expression among the *P. cristatum* transcriptomes. For the RETC, 4.4% and 13.3% of the genes found in the assembly were significantly upregulated in Reduced and Depleted, respectively (Table 2, Supplementary materials Table S5). Interestingly, each complex of the RETC was represented by a gene that was significantly DE in Depleted but not Reduced, illustrating a more profound change in Depleted. The central carbon metabolism of *P. cristatum* contained complete pathways for the tricarboxylic acid (TCA) cycle and glycolytic pathways. Among the transcripts involved in the TCA cycle, 15.2% and 25.8% were DE in Reduced and Depleted, respectively (Table 2). The genes of four of the five key enzymes controlling the TCA cycle—citrate synthase, aconitate hydratase, oxoglutarate dehydrogenase, and succinate dehydrogenase [62]—were upregulated under nutrient restrictions, particularly in the Depleted treatment. The pentose phosphate pathway was similarly DE in both Reduced and Depleted with 23.2% and 24.6% of the pathway, respectively, 80% of which were downregulated. For glycolysis, 16.9% and 27.4% of the genes were DE in Reduced and Depleted, respectively, with most genes downregulated. However, the few upregulated transcripts (*pfkA, PK, frmA, ALDH*) corresponded to key enzymes whose activity affects the rate of glycolysis, or the conversion of pyruvate to acetate for entry into the TCA cycle as acetyl-CoA, a rate-limiting substrate [63].

### Differential expression of genes involved in nutrient uptake and assimilation

Nitrogen metabolism was represented by the downregulation of genes encoding transporters (*NRT*, *pstN*) and enzymes involved in the assimilation of NOx (*nirA, norB*), but the upregulation of genes involved in amino acid cycling (*fmdS, GDH2, GLT1;*Supplementary materials Table S6*)*. In contrast, 20% of phosphate-associated transporters had increased expression levels in both nutrient restrictions, including a sodium-dependent phosphate cotransporter (*SLC34A*), known to be upregulated in phosphate-deprived diatom cells [64] and a substrate-binding subunit of the high-affinity phosphate transport system (*pstS*). Furthermore, alkaline phosphatase D (*phoD*), involved in the hydrolysis of dissolved organic phosphate, was upregulated in both nutrient restrictions (Supplementary materials Table S6).

## Discussion

**Figure 5:**
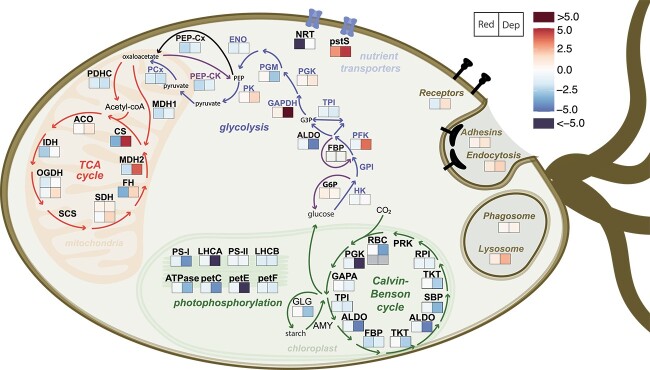
Summary diagram of the pathways affected by differential gene expression patterns in *P. cristatum* under reduced nutrient and depleted nutrient conditions. Squares display the level of up- or downregulation compared to the replete reference transcriptome. The color scale reflects the log2 fold-change values obtained with DESeq2. Under gene groups lysosome, phagosome, endocytosis, and receptors, squares represent the average log2 fold-change value of all up-regulated genes in that category. For gene groups PS-I, PS-II, LHCA, and LHCB, squares represent the average log2 fold-change value of all DE genes. Data represent the means of five biological replicates. Fold-change values, gene IDs and acronyms for the enzyme/protein names are referenced in Supplementary Table S5. “Red”, nutrient reduced condition; “Dep”, nutrient depleted condition.

Our study showed that the increased bacterivory by the prasinophyte *P. cristatum* under nutrient depletion coincided with the upregulation of pathways involved in ingestion and digestion, as well as a concurrent downregulation of genes involved in photophosphorylation, carbon fixation and the biosynthesis of pigments and starch (Fig. 5). These results suggest a potential reduction in photosynthetic activity while bacterivory increased as the cultures experienced nutrient depletion. Hence our transcriptomic data do not support our initial hypothesis that *P. cristatum* would use bacterial prey as an alternative source of nutrients to maintain photosynthetic carbon fixation as found in other obligate phototroph mixoplankton [13, 39, 40].

As the nutrient restriction treatments were exposed to decreasing concentrations of nutrients over time, feeding frequency increased, allowing cultures to grow at rates similar to the nutrient replete treatment for 16 days. These results corroborate previous findings suggestive of inducible phagotrophy [17] in members of the pyramimonadales [27, 65]. Furthermore, the transcriptional responses reflected the physiological changes observed in the growth and feeding experiments. Cultures in f/20 showed progressively stronger gene expression changes in response to decreasing nutrient concentrations, as growth persisted in Depleted beyond Day 11. The greater differential expression of photosynthetic and phagotrophic pathways in Depleted reflected the drastic increase in feeding frequency observed at Day 16 compared to Day 11. Our comparative transcriptomic study therefore captured the shift in gene expression as *P. cristatum* adjusted the relative contribution of phototrophy and phagotrophy to its metabolism*.*

### Comparing the transcriptomic behavior of *P. cristatum* to other constitutive mixoplankton

The transcriptional response of *P. cristatum* to low nutrient concentrations is distinct from that of other constitutive mixoplankton taxa that have been characterized to date. The downregulation of genes involved in photosynthetic metabolic pathways that occurred during increased feeding frequency seems comparable to that of the chrysophyte *Ochromonas* BG-1 [21]. However, the latter is a primarily phagotrophic algae and only relies on photosynthesis for carbon assimilation when prey are limiting growth [16]. *Ochromonas* BG-1 grows in the dark [21], which *P. cristatum* and other prasinophytes are not capable of [12, 27, 28, 66].

Even compared to other obligately photosynthetic taxa, *P. cristatum*’s transcriptional response to low nutrient concentrations is distinct. For example, the dinoflagellate *Prorocentrum shikokuense* upregulates genes involved in the Calvin-Benson cycle, which physiologically transpires as an increase in autotrophic carbon fixation, despite increased bacterivory to compensate for nitrogen limitation [39]. The haptophyte *P. parvum* also uses bacterivory to obtain nitrogen but relies on photosynthetic carbon fixation for growth [13] and likely only assimilates prey carbon for cell maintenance [20]. While one prior study investigated the transcriptomic responses of two prasinophyte species—*Micromonas polaris* and *Pyramimonas tychotreta*—to reduced nutrient concentrations [33], comparing their results with ours regarding changes in gene expression is problematic for multiple reasons. Firstly, the authors used the fold-change differences instead of our more conservative *p*-value approach in identifying DE genes and might therefore have overestimated their number. In addition, inconsistencies among the replicate *P. tychotreta* transcriptomes under the same condition suggest an unreported data quality issue, such as contamination. Adding to the complexity, a recent study [35] shed doubt on the capacity of *M. polaris* to feed on bacteria. Consequently, further investigations of prasinophyte bacterivores are necessary to reconcile these disparities and uncertainties before comparisons can reliably be made with our data. To sum up, the transcriptional downregulation of photosynthetic pathways in *P. cristatum* when nutrient restricted, despite persistent growth, was distinct from that of any other primarily phototrophic mixoplankton reported to date. Such a unique transcriptional response categorizes this prasinophyte as a separate group of constitutive mixoplankton.

### Use of organic carbohydrates and nutrients from prey under low inorganic nutrients

The unexpected downregulation of photosynthetic genes displayed by *P. cristatum* as bacterivory increased could be attributed to the availability of organic carbohydrates from ingested prey. Indeed, the reduction of photosynthetic processes has been attributed to glucose assimilation in non-phagotrophic green algae. For example, a decline in photosynthetic efficiency and alterations to thylakoid structures, accompanied by decreasing expression levels of genes involved in photosynthesis, were observed for *Chromochloris zofingiensis* during osmotrophic mixotrophy on glucose [67]. In our study, the *P. cristatum* TCA cycle was enhanced in Depleted, in parallel to the continued downregulation of the Calvin-Benson cycle. The concurrent upregulation of key regulatory glycolytic enzymes to stimulate glycolysis, occurring in parallel to the suppression of gluconeogenesis and the pentose phosphate pathway, indicates the activation of catabolic processes in *P. cristatum*.

In addition, the transformation of key TCA cycle intermediates such as oxaloacetate and ⍺-ketoglutarate may be accelerated, due to the upregulation of citrate synthase and ⍺-ketoglutarate dehydrogenase, respectively. Anaplerotic steps of the TCA cycle, leading to the biosynthesis of the amino acids aspartate, glutamate, and their derivatives were therefore likely being bypassed. Such a skewing of the metabolic balance toward catabolic processes and away from anabolism suggests that *P. cristatum* was incorporating organic carbohydrates derived from prey into its carbon metabolism, which could explain the downregulation of genes involved in carbon fixation [68]. Phago-mixotrophic prasinophyte lineages probably retain such regulation shared with their osmo-mixotrophic relatives among the chlorophyta to control the use of photosynthesis when organic carbohydrates from prey are present inside the cell and to balance energy expenditure.

In Reduced and Depleted, phosphate and nitrate concentrations were at detection thresholds confirming that *P. cristatum* cultures were exposed to nutrient restrictions. Previously observed autotrophic responses to similar nutrient restrictions indicate that algae tend to decrease carbon fixation and chlorophyll biosynthesis while increasing the production of lipids and scavenging for limiting nutrients [67, 68]. By contrast, lipid metabolism in *P. cristatum* seemed to shift to lipid degradation (Supplementary materials), another catabolic process. Considering that the N:P ratios to which *P. cristatum* was exposed in Reduced (1.75 ± 2.06) and Depleted (5.95 ± 10.2) were below the Redfield ratio (16:1), it was expected that inorganic nitrogen would be the limiting nutrient. However, the downregulation of genes involved in nitrate uptake and assimilation during nutrient restrictions (Supplementary materials Table S6), contrasts with reactions of non-phagotrophic phytoplankton such as the diatom *Phaeodactylum tricornutum* and the chlorophyte green alga *Chlamydomonas reinhardtii*, which tend to upregulate NOx transporters as soon as nitrogen becomes limiting [67, 69].

This opposite response in *P. cristatum* could be because the mixoplankton was getting amino acids from its prey [70, 71]. The capacity to obtain organic forms of nitrogen through bacterivory, allowing it to maintain growth rates in f/20 similar to those of f/2 despite this stark decrease in available nitrate, could have mitigated a stress response affecting the expression of genes involved in nitrogen transport. In contrast, phosphate transporter genes were significantly upregulated in both Reduced and Depleted conditions, illustrating increased efforts to take up inorganic phosphate as the resource was depleted. Additionally, the upregulation of alkaline phosphatase indicates that *P. cristatum* was scavenging phosphorus from dissolved organic matter, further suggesting that the alga was phosphate limited [72], despite feeding. *P. cristatum* might have been compensating for a lower bioavailability of bacterial phosphorous compared to nitrogen, perhaps due to a lower assimilation efficiency by the algae’s metabolism. While we did not quantify the uptake of nutrients from prey and inorganic and organic nutrients, our results suggest that in low-nutrient environments, *P. cristatum* relies on all forms of phosphorous for growth.

### Characterizing phagotrophy in *P. cristatum*

Although the process of photosynthesis has been extensively studied at the molecular level, the understanding of phagocytosis remains limited. This is because the proteins involved are not conserved, are not specific to phagocytosis, and are often involved in other cellular processes [32, 50, 73]. Most of the knowledge on phagocytosis comes from what is known about the immune system of mammals and insects [74, 75]. However, recent studies on phagocytosis in unicellular eukaryotes [32, 50, 71, 76, 77] have provided new information, showing that not all phagocytic lineages share the same proteins involved in the process [32]. Our study contributes to the growing knowledge on genes involved in phagotrophy that could serve as quantifiable markers for mixotrophic activity, providing complementary approaches to the study of mixoplankton in their natural environments [78].

The predictive model [32] highlighted key phagocytotic genes in *P. cristatum*. In particular, *BBS2*, upregulated in actively feeding *P. cristatum* cultures, codes for a subunit of the Bardet–Beidl Syndrome complex (BBSome) involved in the transport of cilia membrane proteins, affecting the motility and sensory functions of cilia and possibly flagella [79]. Hence, the BBSome might have a key role in flagellar movements necessary for prey capture in prasinophytes. Cathepsins, cysteine proteases, have also been reported as essential to phagotrophic activity in small-sized planktonic eukaryotes, making up the most diverse and abundant transcripts found in marine stramenopile environmental transcriptomes [50]. Our study reveals a more complex dynamic in *P. cristatum*, with the downregulation of certain cathepsins alongside the upregulation of others, likely related to their respective function in prey digestion and substrate specificity [80], compared to other specialized processes such as autophagy [81] or protein processing [82]. Future investigations into these few pivotal “phagocytotic” genes would help refine the description of this process in prasinophytes, and likely in other mixoplankton.

Members of the Pyramimonadales, such as *P. cristatum* and *Cymbomonas tetramitiformis*, possess a feeding apparatus, located near the flagellar pit and consisting of a mouth-like opening connected by a tubular channel to a large vacuole [27, 83–85]. It has been suggested that these structures are utilized by the cell to internalize and digest bacterial prey [27]. As such, these organisms might not use the same cellular processes as amoeboid or mammalian cells to engulf bacterial prey. Nonetheless, we found upregulation of genes involved in digestion processes as well as vesicle formation and trafficking. Ciliates, who also have a defined mouth-like structure for feeding, use a form of vesicular trafficking to deliver vacuolar ATPases and digestive enzymes to the food vacuole at the base of the oral apparatus [86]. Pyramimonadales might have a similar process to support the prey digestion taking place in the permanent food vacuole. Furthermore, recognition mechanisms might be shared with the amoebozoan and opisthokont species, as indicated by the presence of a folate receptor involved in predation in *Dictyostelium* [76, 87] as well as other G-protein coupled receptors that were tightly regulated in *P. cristatum*. Similarly, the increasing upregulation of adhesion proteins between the Reduced and the Depleted conditions, suggests that *P. cristatum* required different surface adhesive molecules as nutrients became more limiting and that it relied more heavily on bacterivory.

## Conclusion

Our results suggest that obligate phototroph constitutive mixoplankton like *P. cristatum* are likely to be more heterotrophic than suspected, particularly in high-light/low-nutrient waters, such as the subtropical gyres that constitute the largest oceanic biome. Subtropical gyres have been correlated with elevated proportions of mixoplankton exhibiting high ingestion rates [4, 88, 89]. This has potential consequences on net CO_2_ fixation, as well as carbon transfer through the food chain and its export to the deep ocean [7]. The ratio of bacteria to phytoplankton cell abundance tends to increase along gradients of nutrients from coastal waters to oligotrophic open-oceans, for instance reaching values of 1300 to 2289 in the subtropical and tropical North Atlantic [34]. This provides an abundant alternative source of nutrients for mixoplankton, most likely greater than that in our *P. cristatum* cultures characterized by average ratios of 13:1. A recent metatranscriptomic study of mixoplankton communities from a nutrient-limited subtropical gyre revealed their capacity to modulate photosynthesis against bacterivory as a function of nutrient availability [90], suggesting that the nutritional strategy used by *P. cristatum* might be more widespread than previously expected. These results caution us against modeling constitutive mixoplankton as a monolithic group; the physiological dynamics of mixoplankton in natural environments might vary based on the dominant taxa present, affecting our capacity to predict their impact on local conditions, such as pH [91].

In summary, our study provides novel insights into a new category of phago-mixotrophy. We found that under nutrient depletion, distinct from other primarily phototrophic constitutive mixoplankton, *P. cristatum* downregulates the expression of genes involved in photosynthesis, likely reducing its photosynthetic activity, while increasing its bacterivory. This dynamic physiology reflects the delicate balance of energetic tradeoffs between phototrophy and heterotrophy inherent to mixoplankton [19]. When low inorganic nutrient concentrations trigger phagotrophy, *P. cristatum* likely redirects cellular resources toward a digestive machinery and the energetic tradeoff dictates a halting of autotrophic processes and a new reliance on prey as a source of carbon in addition to the limiting nutrient. The downregulation of photosynthesis, concurrent with the observed increase of bacterivory under optimal light conditions, carries important implications for mixoplankton ecology and oceanic carbon cycling [34, 89, 92]. Hence, our findings warrant further validation through complementary approaches, including quantitative reverse transcription polymerase chain reaction assays of key genes identified in this study across a wide range of physiological conditions, quantitative proteomics experiments, and measurement of carbon fixation rates with isotopes to substantiate changes in the metabolic activity suggested by our results. Moreover, to be able to generalize, future investigations should explore whether other members of the Pyramimonadales exhibit comparable or different metabolic adaptations.

## Supplementary Material

supplementary_Material_ycae083

## Data Availability

The algal strain used in this study can be obtained from the Microbial Culture Collection at the National Institute for Environmental Studies (NIES collection, Tsukuba, Japan). Raw sequencing data are available in the NCBI short read archive database (SRA) under the following accession numbers: SRR29192187–SRR29192181. The de novo transcriptome assembly has been deposited at DDBJ/EMBL/GenBank under the accession GKVK00000000. The version described in this paper is the first version, GKVK01000000. Expression data are available as GSE268495 in the NCBI Gene Expression Omnibus (GEO) database.
