# Supplementary material for: Transcriptomics reveal a unique phago-mixotrophic response to low nutrient concentrations in the prasinophyte Pterosperma cristatum
Source: ISME Commun. 2024 Jun 14;4(1):ycae083. doi: 10.1093/ismeco/ycae083 (PMC11217555; doi:10.1093/ismeco/ycae083)
Supplement: supplementary_Material_ycae083 [file supplementary_material_ycae083.zip › SupplMaterialsFigures_ISMEcomm_Corr.pdf]

Supp. Materials and Methods

Growth in nutrient availability experiments

To prepare experimental treatments, a 600 ml *Pterosperma cristatum* NIES626 culture was maintained as per typical growth conditions in f/2 (without silicate) at 17˚C under roughly 80 µE m^{-2} s^{-1} irradiance on a 12 hour light:dark cycle. Culture growth was monitored by regular sampling for *in vivo* fluorometry. At mid exponential phase, the culture was concentrated by dividing volumes into 14 separate 50 ml Falcon© tubes (FisherScientific) and centrifuging at 500 g for 3 minutes. The supernatant was removed from each tube and 1mL of artificial seawater (ASW) was used to resuspend all 14 pellets. The density of the concentrated cell suspension was determined via flow cytometry. Aliquots were transferred to 50-ml Erlenmeyer flasks containing 25 ml of f/2 or f/20 growth medium to reach a final density of 1.8 x 10^5 cells in all flasks. Every treatment was represented by 5 replicates. Nutrient replete f/2 medium (without silicate) was prepared as described in Guillard (1975) in 33 psu ASW. The f/20 medium was prepared by diluting f/2 medium 1:10 in 33 psu ASW. Experimental cultures were then grown for 18 days, during which cell density and bacterivory were monitored to identify the appropriate time to collect samples for RNA.

Preparation of fluorescently labeled bacteria

Fluorescently labeled bacteria (FLB) were prepared according to Bock et al. (2021). Briefly, cultures of *Pelagibaca bermudensis* were grown to mid-exponential growth phase in 10 mL ASW enriched with glucose (0.2% w/v) and yeast extract (0.5% w/v). On the day prior to feeding experiments, bacterial cultures were pelleted via centrifugation. The mass of the pellet was then weighed and diluted to a final density of 1 mg/mL. A 5 µL aliquot of CellTracker Green CMFDA (Thermo Fisher Scientific, Waltham, MA) suspended in 10 µL DMSO was then added to a 1-mL aliquot of the diluted cell suspension. This volume was then incubated in a water bath at 35˚C for three hours. Following incubation, FLB were washed five times via centrifugation and resuspended in 1 mL ASW. FLB were then stored overnight under normal growth conditions. On the day of feeding experiments, FLB were washed again. Supernatant from the final wash step was filtered < 0.2 µm and saved for use as a negative control (see below).

Statistical analysis of bacterivory measurements

To test for significant differences in variation between experimental conditions, a two-way ANOVA was carried out using R base, followed by a Tuckey's test to determine the pairwise significant differences. To evaluate differences in feeding frequencies, replicate per_{fed} values were pooled for each condition (Replete, Reduced, and Depleted). Multivariate regressions were then performed comparing per_{fed} to the time elapsed since inoculation with FLB, including experimental condition (Control, Replete, Reduced, and Depleted) as a categorical variable. To mitigate bias due to non-linear changes in the number of cells exceeding feeding thresholds, only measurements from the first 30 minutes of feeding experiments were included in the regressions. To determine whether feeding frequencies were significantly different across experimental conditions, pairwise t-tests were performed to evaluate significant differences between interaction terms in the regression model (e.g., regression coefficients associated with the combined effect of experimental condition and time since inoculation). A significance threshold of 0.05 was used for all comparisons.

RNA collection, extraction, and sequencing

To preserve total RNA until extraction, 20 mL culture samples were transferred to 50 mL Falcon tubes containing 20 mL RNA*later* Stabilization Solution (Invitrogen), mixed by inverting several times and stored at 4°C for 1 week, as per manufacturer instructions. The mixture of RNA*later Stabilization Solution* and culture was then gently filtered onto 0.8 µm 47 mm polycarbonate (PCTE) filters over a 47 mm polyester (PETE) drain disc (Sterlitech Corporation). The filters were placed into individual Petri dishes, and incubated with 1 mL of Trizol Reagent (Invitrogen) for lysis of algal cells during 5-10 min. After this, the volume of Trizol was used to flush each filter several times and transferred to a 1.7 mL tube. Chloroform (0.2 mL) was added, thoroughly mixed with the lysate, and incubated for 2-3 min. After centrifugation at 4°C for 15 min. at 12,000 g, the clear aqueous phase was carefully transferred to a fresh tube (~400-500 µL) and mixed with an equal volume of 70% ethanol before loading onto a PureLink RNA mini kit (Invitrogen) column. Washing of the on-column RNA was conducted as per manufacturer instructions, followed by an elution step in 50 µL of RNase-free water provided with the kit. The RNA extracts were then quantified with a Qubit RNA HS assay kit (Invitrogen) and subsequently sent to GeneWiz for polyA selection with a TruSeq library preparation and sequencing on an Illumina HiSeq 4000 with a 2x 150bp format.

Bioinformatic transcriptome analysis

The Illumina reads were quality checked with FastQC, prior to *de novo* assembly with rnaSPAdes (Bushmanova et al., 2019) using three replicates each from the Replete, Reduced and Depleted conditions. Transcripts that correspond to the co-cultured prokaryotes were identified by BLASTn analysis with NCBI-BLAST+ (v2.12; (Camacho et al., 2009)) against the bacteria taxids from NCBI nt database v5_08Jun2018, and removed before re-assembly using the hard filtered option to further remove transcripts < 300bp. Redundant transcripts were collapsed with CD-HIT-EST (v4.8.1; Li & Godzik, 2006) for a final transcriptome model. The full transcriptome assembly consisted of 72,305 coding regions or transcripts identified with TransDecoder (v.5.5.0; Haas; https://github.com/TransDecoder/TransDecoder). The Replete treatment was represented by a transcriptome of 52,967.2 (± 3,042.3) genes, while Reduced and Depleted treatments were composed of 53,508.6 (± 3,426.4) and 52,751.8 (± 3,098.8) genes, respectively. Transcripts were annotated with the KEGG ontology using the GhostKOALA online tool (Kanehisa et al., 2016). Annotated transcripts were then cross-referenced to KEGG Pathways through the KEGGREST package in R (Tenenbaum & Volkening, 2021). Reads were mapped and counted using Salmon (v1.6; Patro et al., 2017).

Supp. Results

Differential expression of lipid and amino acid metabolic pathways

Lipid metabolism was 18.8% and 30.6% differentially expressed under Reduced and Depleted, respectively (Supp. Table S6). Among the glycerolipid and glycerophospholipid metabolism, upregulated genes were involved in degradation and anaplerotic transformations. In contrast, the production of fatty acids, including polyunsaturated fatty acids was mostly downregulated. Amino acid metabolism was 22.5% and 31.7% differentially expressed in Reduced and Depleted, respectively (Supp. Table S6). Upregulation in nutrient restrictions pertained to genes involved in the release of ammonium (*ASRGL1, nadB, DAO, AOC3, cuyA, davA*) or glutamine and ATP (*ACAD8, cysE, ISS1, serC, GLYK, proB*) from amino acid catabolism.

Supp. Discussion

Methodological limitations:

The use of transcriptomics comes with intrinsic assumptions that the process is regulated at the transcriptional level and that all cells present a homogenous response. However, increased expression of phagocytotic genes observed in *Dyctiostelium,* correlated with predatory phagocytosis (Sillo et al., 2008), guarantees that at least part of phagocytosis is regulated via transcription. Asynchrony within cultures can also be a source of methodological noise in transcriptomic studies. In our pure *P. cristatum* cultures, not all individual cells were feeding at any given time, suggesting that the switch to predation was likely asynchronous. As RNA was collected from a mixture of algal cells at different stages of the metabolic switch, population heterogeneity (Ma et al., 2021; Ogata et al., 2021) might translate as attenuated expression signals and low significance for feeding-specific genes in the total culture transcriptome. Notably, the higher proportion of feeding *P. cristatum* cells in Depleted conditions was accompanied by a more pronounced transcriptional signal.

Supp. References:

Bock, N., Charvet, S., Burns, J., Gyaltshen, Y., Rozenberg, A., Kim, E., & Duhamel, S. (2021). Experimental identification and in silico prediction of bacterivory in green algae. *ISME J*. https://doi.org/10.1038/s41396-021-00899-w

Bushmanova, E., Antipov, D., Lapidus, A., & Prjibelski, A. D. (2019). rnaSPAdes: a de novo transcriptome assembler and its application to RNA-Seq data. *GigaScience*, *8*(9), 1–13. https://doi.org/10.1093/GIGASCIENCE/GIZ100

Camacho, C., Coulouris, G., Avagyan, V., Ma, N., Papadopoulos, J., Bealer, K., & Madden, T. L. (2009). BLAST+: architecture and applications. *BMC Bioinformatics*, *10*. https://doi.org/10.1186/1471-2105-10-421

Guillard, R. (1975). Culture of phytoplankton for feeding marine invertebrates. In W. Smith & M. Chanley (Eds.), *Culture of Marine Invertebrate Animals* (pp. 29–60). Proceedings - 1st Conference on Culture of Marine Invertebrate Animals Greenport.

Kanehisa, M., Sato, Y., & Morishima, K. (2016). BlastKOALA and GhostKOALA: KEGG tools for functional characterization of genome and metagenome sequences. *Journal of Molecular Biology*, *428*(4), 726–731. https://doi.org/10.1016/j.jmb.2015.11.006

Li, W., & Godzik, A. (2006). Cd-hit: a fast program for clustering and comparing large sets of protein or nucleotide sequences. *Bioinformatics (Oxford, England)*, *22*(13), 1658–1659. https://doi.org/10.1093/bioinformatics/btl158

Ma, F., Salomé, P. A., Merchant, S. S., & Pellegrini, M. (2021). Single-cell RNA sequencing of batch Chlamydomonas cultures reveals heterogeneity in their diurnal cycle phase. *The Plant Cell*, *33*(4), 1042. https://doi.org/10.1093/PLCELL/KOAB025

Ogata, N., Nishimura, A., Matsuda, T., Kubota, M., & Omasa, T. (2021). Single-cell transcriptome analyses reveal heterogeneity in suspension cultures and clonal markers of CHO-K1 cells. *Biotechnology and Bioengineering*, *118*(2), 944–951. https://doi.org/10.1002/bit.27624

Patro, R., Duggal, G., Love, M. I., Irizarry, R. A., & Kingsford, C. (2017). Salmon provides fast and bias-aware quantification of transcript expression. *Nature Methods*, *14*(4), 417–419. https://doi.org/10.1038/nmeth.4197

Sillo, A., Bloomfield, G., Balest, A., Balbo, A., Pergolizzi, B., Peracino, B., Skelton, J., Ivens, A., & Bozzaro, S. (2008). Genome-wide transcriptional changes induced by phagocytosis or growth on bacteria in Dictyostelium. *BMC Genomics*, *9*, 1–22. https://doi.org/10.1186/1471-2164-9-291

Tenenbaum, D., & Volkening, J. (2021). *KEGGREST: Client-side REST access to the Kyoto Encyclopedia of Genes and Genomes (KEGG)*.

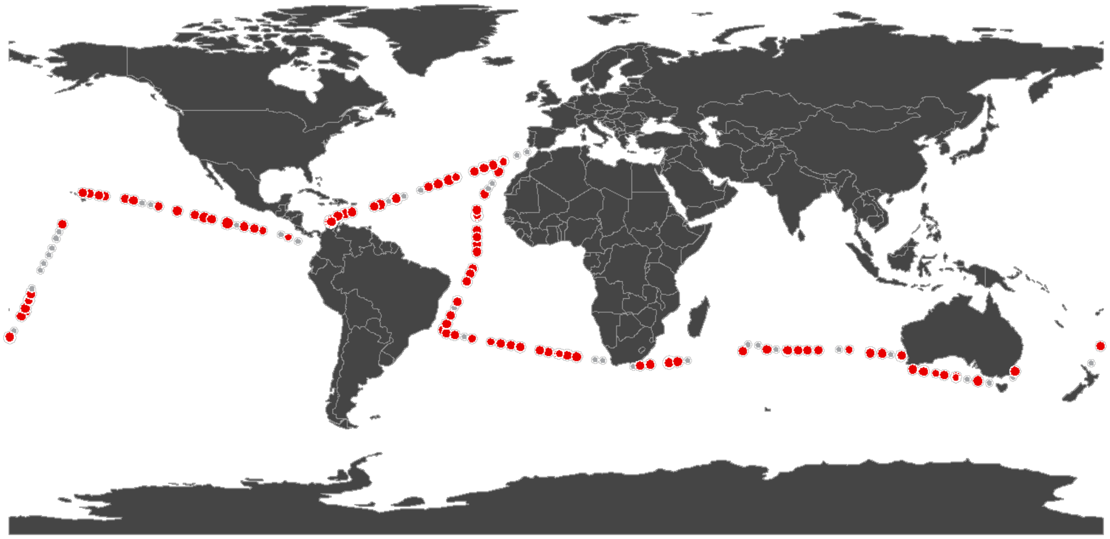

Supp. Fig. S1: Global distribution of *P. cristatum* NIES626. Map created using the MicroMap online tool (Obiol et al., 2021). Red dots represent stations with at least one Pterosperma cristatum ASV. Gray dots, stations without any *P. cristatum* amplicon sequence variants.

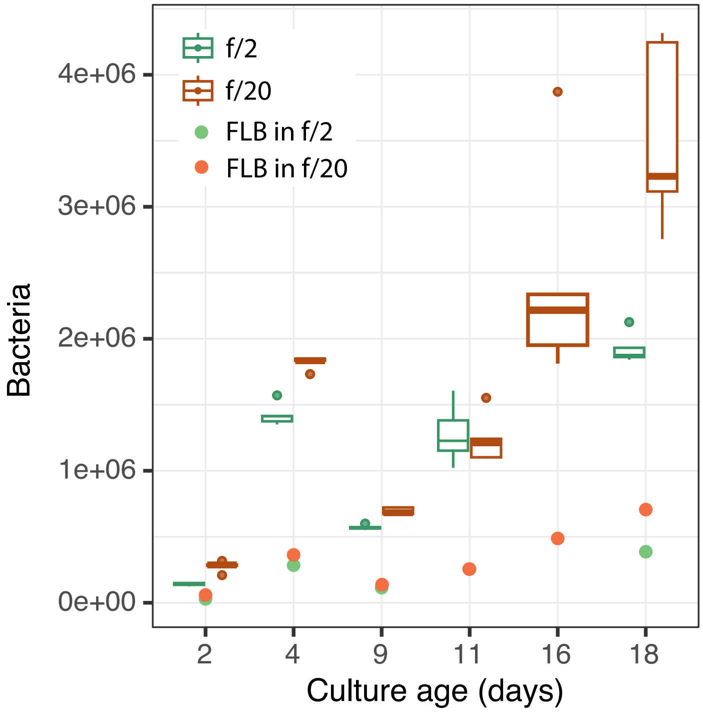

Supp. Fig. S2: Abundance of ambient bacteria measured in the 0.5mL aliquots of *P. cristatum* cultures used for feeding experiments at different time points**.** Boxplots indicate the median, 95th and 25th quartiles, for all replicates (n=5). Outliers are shown as small dots. The number of fluorescently labeled bacteria (FLB) added to each tube for feeding experiments is indicated by thicker dots. Note that FLB were added to correspond to 20% of the ambient bacterial cell count for each aliquot of *P. cristatum* culture.

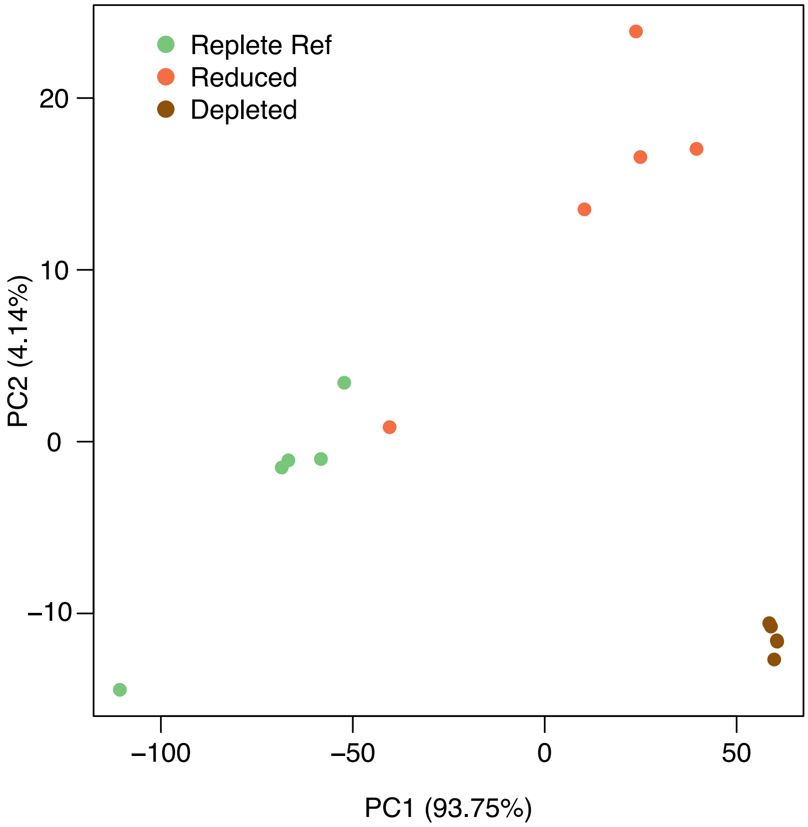

Supp. Figure S3: Differences between the *P. cristatum* transcriptomes from each condition. This principal component analysis, based on a Spearman distance matrix, illustrates the differences among the transcriptomes obtained from the replete reference, the nutrient reduced (Reduced) and the nutrient depleted (Depleted) conditions. Each dot represents a replicate transcriptome (n=5).

Supp. Figure S4: MA plots illustrating the differential gene expression between (A) the nutrient reduced condition (NR) and replete reference (RR), between (B) the nutrient depleted condition (ND) and replete reference and between (C) the ND and NR. Dots in red correspond to genes with a significant differential expression between condition and RR, as determined by DESeq2 using LFC shrinkage, and represented as log2-fold change on the y-axis. Base mean corresponds to the average expression level.

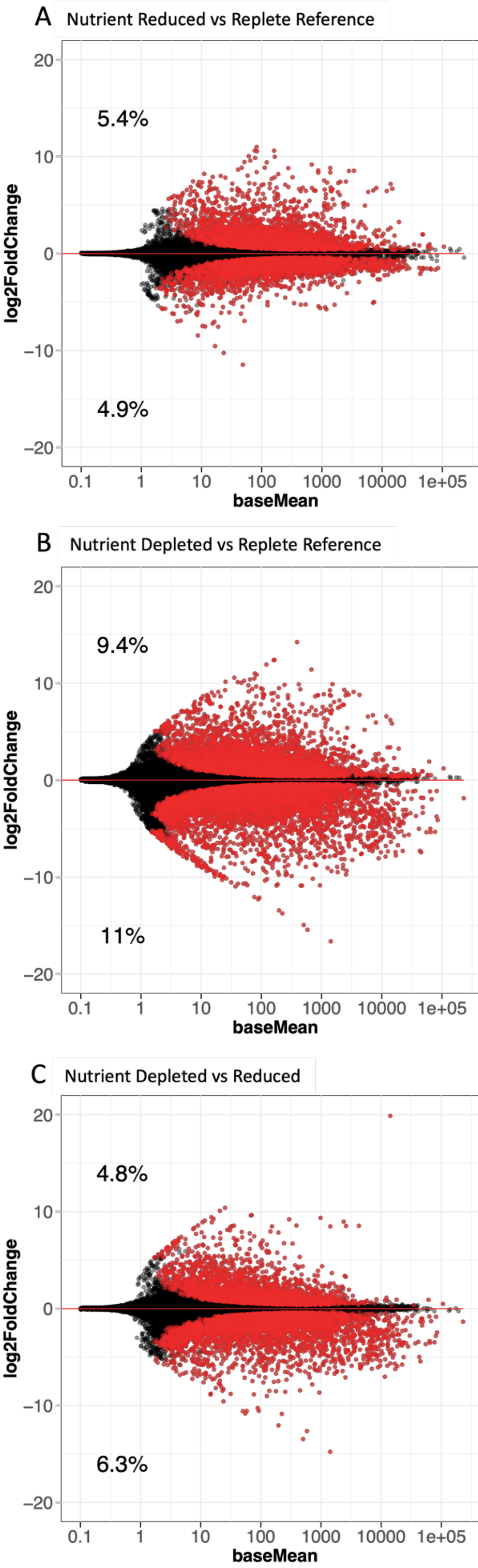

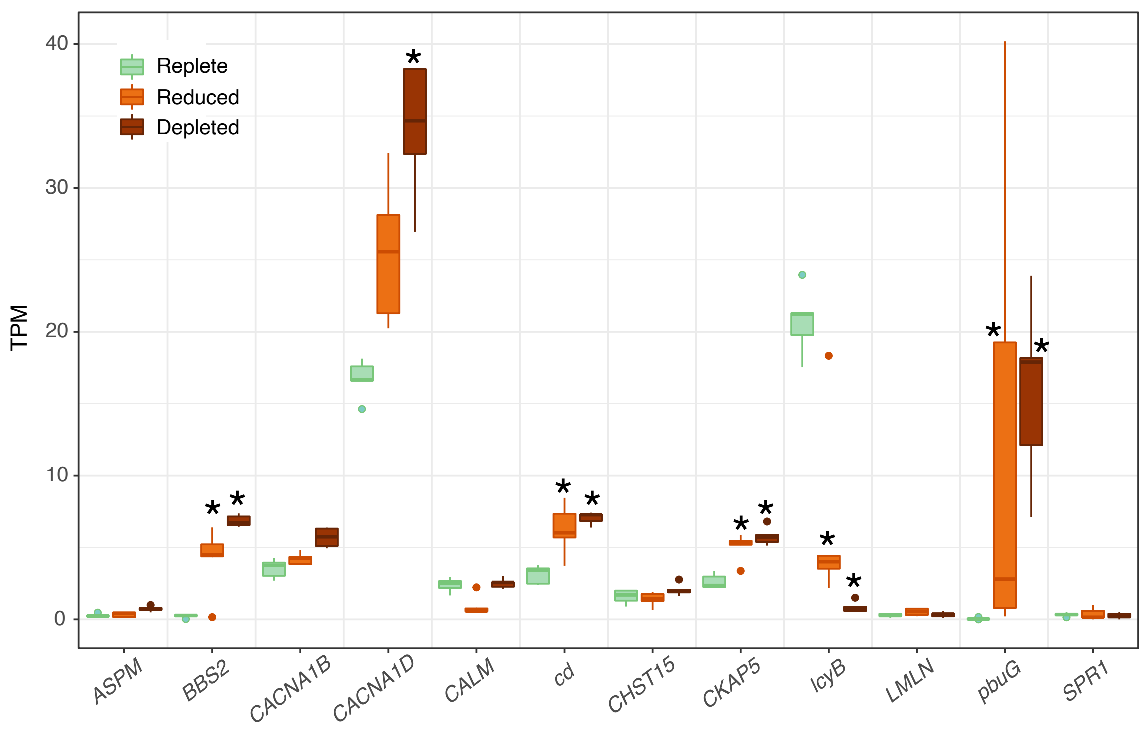

Supp. Fig. S5: Expression levels of genes identified as predictive of phagotrophy by the gene-based prediction model (Burns et al., 2018). Asterix indicate the condition for which the gene was found to be significantly differentially expressed compared to the Replete reference by DESeq2. *ASPM*, abnormal spindle-like microcephaly-associated protein; *BBS2*, Bardet-Biedl syndrome 2 protein ; *CACNA1B*, voltage-dependent calcium channel N type alpha-1B; *CACNA1D*, voltage-dependent calcium channel L type alpha-1D ; *CALM*, calmodulin; *cd*, cyclomaltodextrinase ; *CHST15*, N-acetylgalactosamine 4-sulfate 6-O-sulfotransferase; *CKAP5*, cytoskeleton-associated protein 5 ; *lcyB*, lycopene beta-cyclase; *LMLN*, leishmanolysin-like peptidase; *pbuG*, adenine/guanine/hypoxanthine permease ; *SPR1*, protein SPIRAL1 and related proteins. Replete, replete reference transcriptome; Reduced, nutrient reduced condition; Depleted, nutrient depleted condition.
