# Supplementary material for: Transcriptomics reveal a unique phago-mixotrophic response to low nutrient concentrations in the prasinophyte Pterosperma cristatum
Source: ISME Commun. 2024 Jun 14;4(1):ycae083. doi: 10.1093/ismeco/ycae083 (PMC11217555; doi:10.1093/ismeco/ycae083)
Supplement: supplementary_Material_ycae083 [file supplementary_material_ycae083.zip › SuppTableS1_TranscriptomeStats-Rev.docx]

**Supp. Table S1**: Detailed statistics of the *P. cristatum* transcriptomes. Total transcripts refers to the total counts for the transcriptome assembled *de novo* with representatives from all three sampling conditions (replete reference (RR); nutrient reduced treatment (NR); nutrient depleted treatment (ND)), for a full coverage of genes. Numbers in parentheses refer to standard deviation among the five replicates per sampling condition.

| **Analysis step** | **# of transcripts** |
| --- | --- |
| Total transcripts | 72 305 |
| Transcripts mapped | 72 305 |
| RR | 52 967.2 (± 3 042.3) |
| NR | 53 508.6 (± 3 426.4) |
| ND | 52 751.8 (± 3 098.8) |
| Coding sequences | 52 608 |
| KEGG annotated | 10 543 |
| RR | 7973.8 (± 574.5) |
| NR | 8018.2 (± 586) |
| ND | 7608.4 (± 777.3) |
