# Supplementary material for: Transcriptomics reveal a unique phago-mixotrophic response to low nutrient concentrations in the prasinophyte Pterosperma cristatum
Source: ISME Commun. 2024 Jun 14;4(1):ycae083. doi: 10.1093/ismeco/ycae083 (PMC11217555; doi:10.1093/ismeco/ycae083)
Supplement: supplementary_Material_ycae083 [file supplementary_material_ycae083.zip › SuppTableS2_FeedingStats-Rev.docx]

**Supp. Table S2**: Pairwise differences of interaction terms in multiple regressions comparing per_fed_ to time since incubation and experimental conditions. All differences are described by the t-score (p-value). Statistically significant differences are in bold. “control”, supernatant unfed control

|  | **control** | **Day 11** | | **Day 16** |
| --- | --- | --- | --- | --- |
|  |  | **f/2** | **f/20** | **f/20** |
| **0** | 0.081  (p = 0.94) | 0.15  (p = 0.14) | **3.21 (p = 0.002)** | **15.8**  **(p < 2 x 10^-16^)** |
| **SN control** |  | 0.87  (p = 0.39) | 1.93  (p = 0.06) | **9.64**  **(p = 2 x 10^-14^)** |
| **f/2 – Day 11** |  |  | 1.83  (p = 0.07) | **15.2**  **(p = 1 x 10^-23^)** |
| **f/20 – Day 11** |  |  |  | **13.4**  **(p = 1 x 10^-20^)** |
